# Acute and Subacute Toxicity of Fluorescent Gold Nanoclusters Conjugated with α-Lipoic Acid

**DOI:** 10.3390/nano12213868

**Published:** 2022-11-02

**Authors:** Yun-Fang Chen, Chun-Chieh Hsu, Ching-Hu Chung

**Affiliations:** Department of Medicine, Mackay Medical College, New Taipei City 252, Taiwan

**Keywords:** acute toxicity, subacute toxicity, fluorescent gold nanoclusters

## Abstract

Fluorescent gold nanoclusters conjugated with α-lipoic acid (FANC) is a promising biocompatible fluorescent nanomaterial with a high potential for drug development. However, there is still no FANC-related research on toxicology, which is very important for future research and the development of healthy food supplements or drugs. This study uses oral administration of FANC to determine the most appropriate dose range in ICR mice for further evaluation. The *in vivo* acute and subacute toxicity study was conducted by oral administration of FANC to male and female ICR mice. Animal survival, body weight, daily food consumption, hematological profile, organ coefficient, serum biochemistry profile, and histopathological changes were analyzed. FANC did not show any form of morbidity or mortality at acute and subacute toxicity in both male and female ICR mice. Animal behavior, daily food consumption, hematological profile, organ coefficient, and histopathology showed no treatment-related malignant changes at single and repeated doses. Furthermore, serum glutamic-oxaloacetic transaminase (GOT), glutamic-pyruvic transaminase (GPT), lactate dehydrogenase (LDH), blood urea nitrogen (BUN), and creatinine (CRE) levels showed no significant malignant changes, which indicated that FANC does not cause liver and renal damage. The only change observed in this study was the change in body weight. The body weight of the FANC-treated group was slightly decreased in female mice but increased in male mice; however, the body weight decreases were below the threshold of concern, and there was no dose–response effect. In conclusion, no observed adverse effect level (NOAEL) in repeated doses was considered in 20 μM/100 μL/25 g male and female ICR mice.

## 1. Introduction

The properties of gold nanoparticles, such as stability, bioinert, and low toxicity, make them suitable for developing healthy food supplements or medicines as a drug delivery system [1]. Applications of gold nanoparticles are various, including drug delivery, photothermic therapy, imaging, and targeting (conjugated with antibodies) [2]. Orally administered gold nanoparticles can be absorbed in the circulation and then arrive in the target organ [3]. Gold nanoparticles make images clearer to help coronary angiography with very low toxicity in clinical applications [4]. A previous study also discovered that endothelial progenitor cells labeled by FANC did not destroy the angiogenic potential and demonstrated good biocompatibility [5].

However, inorganic nanoparticles are difficult to biodegrade and induce long-term toxicity, which hinders their application in clinical practice [6]. Studies have found that particles smaller than 5.5 nm were eliminated by the kidney rapidly and efficiently [6,7], which allows them to be used safely in the clinic. A recent report showed that the renal clearance rate of glutathione-protected gold nanoclusters (2.1 nm) was maximum at 2 h after injection, and only 6% of gold could be detected in mice at 28 days [7]. For further development of nanoparticle applications, assessing their potential toxicity is necessary to avoid adverse effects on humans and the environment [8]. A previous study analyzed the cytotoxic effects of dihydrolipoic acid-capped gold NCs on the normal human hepatic cell line (L02) and human hepatoma cell line (HepG2), and results showed that the proliferation of HepG2 cells was significantly inhibited, but it did not affect L02 cells [9].

α-Lipoic acid (LA), a type of thioctic acid, is naturally synthesized by some plants and animals, including humans [10]. Endogenous LA acts as a cofactor for mitochondrial enzymes and other multienzyme complexes, including those of branched-chain α-keto acid, pyruvate dehydrogenase, α-ketoglutarate dehydrogenase, and glycine decarboxylase [11,12]. The reduced form LA, dihydrolipoic acid (DHLA), is a capping ligand utilized in the preparation of hydrophilic nanoparticles for cellular labeling and tracking, including the detection of tracers in embryonic development investigation [13,14,15,16]. Recent studies have shown that LA and DHLA function in various cells to block or prevent oxidative stress-induced apoptosis but promote apoptosis in several cancer cell lines [17,18,19,20,21,22]. Our recent study reported that DHLA at concentrations of 50–100 μM triggered apoptosis of mouse embryonic stem cells (ESC-B5) and had no cytotoxic effects at 0–25 μM [23]. The most important is LA which has anti-oxidant and anti-inflammatory activities. This acid antagonizes nanomaterials, inducing inflammatory responses and oxidative damage toxicity [24]. This is mainly through increased GSH, decreased IL-6, and chelation of metal ions [24]. Therefore, FANC, which we used in this study, carries LA and may prevent the toxicity induced by gold nanoclusters.

In the previous studies, co-workers in our team developed a one-pot synthetic strategy to synthesize water-soluble DHLA-capped gold nanoclusters (DHLA-Au NCs), which are promising imaging agents for biomedical and cellular applications [5,15]. In the cytotoxic test, cells treated with 1 nM 13 nm gold significantly decreased proliferation and ATP bioenergy, but 100 nM DHLA-Au NCs treatment only displayed minimal changes [25]. DHLA-Au NCs can also attenuate cellular senescence, attenuate mitochondria-derived oxidative stress, and decrease the production levels of the inflammatory cytokines induced by lipopolysaccharide both in vitro and *in vivo* [25]. A previous study also indicated that FANC-labeled endothelial progenitor cells preserved intact angiogenic potential, demonstrating that FANC is highly biocompatible [5].

FANC is a promising biocompatible fluorescent nanomaterial and has a high potential for drug development for ameliorating oxidate stress and/or aging, treatment of hypercholesterolemia, or conditions associated with hypercholesterolemia. However, the safety and toxicity of FANC is not fully understood yet. Thus, the present study focuses on the safety assessment of FANC through acute and subacute toxicity evaluations by oral administration using mice as animal models.

## 2. Materials and Methods

### 2.1. Synthesis of FANC via a One-Pot Synthetic Strategy

The FANC used in this study was produced by GOLDRED NANOBIOTECH CO., LTD. (Taoyuan, Taiwan). FANCs were synthesized by a one-pot synthetic according to the methods previously described [5,15]. Briefly, using gold nanoparticles (6 nm) stabilized with didodecyldimethylammonium bromide as material and drops, AuCl_3_ (in DDAB/toluene solution) were added to produce nanoclusters. Then, reduced lipoic acid was added to the ligand exchange through a series of precipitation, centrifugation, reconstitution, and drying methods to remove excess composition. At last, a centrifuge filter (30 kDa molecular weight cutoff) was used to exchange the buffer with the deionized water. The produced FANC product was dark brown in daylight and red fluorescent in UV light. The FANC was a 1.56 ± 0.3 nm diameter gold nanocluster with negative-charged surface modification, water-soluble, and had a high quantum yield (QE∼7%).

### 2.2. Animals and Ethical Statements

Institute of Cancer Research (ICR) mice were used in this subproject and obtained from BioLASCO Taiwan (Taipei, Taiwan). All the experimental protocols regarding animal experiments were approved by the Laboratory Animal Use Committee of MacKay Medicine College (A1080016). All animals received appropriate care as indicated in the Guidelines for Care and Use of Experimental Animals (Canadian Council on Animal Care, Ottawa, 1984). All mice were maintained on breeder chow (Harlan Teklad chow) with food and water available ad libitum. Housing was in standard 28 cm × 16 cm × 11 cm (height) polypropylene cages with wire-grid tops and kept under a 12 h day/12 h night regimen.

### 2.3. Acute Toxicity Test

ICR mice (average weight 25 g) placed in separate cages were randomly divided into five groups and allowed to acclimatize for 1 week. Animals received oral gavage with various concentrations of FANC. Mice were observed at 24 h post-administration for mortality, sacrificed for organ damage monitoring, or further observed for 14 days. Lethal dose, 50% (LD50) values of FANC-feeding animals were measured for 14 days. Toxicity signs were recorded systematically for the first 24 h. After the experiment, surviving mice were examined daily for clinical signs of toxicity for another 14 days. The body weights and food consumption of animals were measured before FANC intake, followed by weekly measurements. On day 15, the surviving animals were sacrificed, and their internal organs, including the heart, lungs, liver, kidney, spleen, adrenal glands, thymus, brain, and sex organs, were excised, weighed, and subjected to a series of pathological examinations. Organ tissues were subsequently fixed in a 10% neutral buffered formaldehyde solution for further histopathological analysis. Blood samples were collected on days 1, 7, and 14 after FANC administration. Blood samples (0.1 mL) were collected into tubes with 3.8% sodium citrate for hematological parameter analysis. On day 14, another blood sample was collected for serum biochemistry.

### 2.4. Subacute Toxicity Test

Subacute tests followed the World Health Organization and Organization for Economic Co-operation and Development guidelines. In the subacute toxicity test, animals were administered orally by gavage with various concentrations of FANC for 14 days. Animals were weighed and observed daily for toxicological signs, physiological and behavioral changes, and mortality. Blood samples were collected on days 1, 7, and 14 after FANC administration for hematological parameter analysis. After the experiment, animals were sacrificed for organ damage monitoring, and blood samples were collected for serum biochemistry and hematological parameter analysis. Organ tissues were subsequently fixed in a 10% neutral buffered formaldehyde solution for further histopathological analysis.

### 2.5. Hematological Parameter Analysis

Hematological analysis (Sysmex KX-21 Hematology Analyzer; Mundelein, IL, USA) was performed using standard techniques for red blood cell count (RBC), hemoglobin (HGB), hematocrit (HCT), mean corpuscular volume (MCV), mean corpuscular hemoglobin (MCH), mean corpuscular hemoglobin concentration (MCHC), red blood cell distribution width (RDW), platelet count (PLT), white blood cell count (WBC), lymphocyte percentage (LYM%), lymphocyte count (LYM#), platelet distribution width (PDW), mean platelet volume (MPV), and platelet large cell ratio (P-LCR) concentrations.

### 2.6. Serum Biochemistry Analysis

Blood samples were biochemically analyzed (Fuji Dri-Chem 4000i; Tokyo, Japan) for levels of creatinine (CRE), blood urea nitrogen (BUN), glutamic-pyruvic transaminase (GPT), glutamic-oxaloacetic transaminase (GOT), and lactate dehydrogenase (LDH).

### 2.7. Statistics

Continuous data are expressed as mean ± standard deviation and are compared with the two-tailed *t*-test. For multiple comparisons, data were analyzed by one-way analysis of variance followed by the Newman–Keuls comparison test. All *p*-values were two-sided, and a *p*-value less than 0.01 was considered significant.

## 3. Results

### 3.1. Acute Toxicity Test

The results of acute toxicity testing of FANC revealed that the oral administration of a single dose (0.6, 2, 6, and 20 μM/100 μL/25 g mice) did not show any sign of morbidity or mortality in the treated animals during the 14 days. All mice survived during the 14 days observation period, and the mice exposed also showed no behavioral changes at the doses administered during the treatment period.

**Figure 1:**
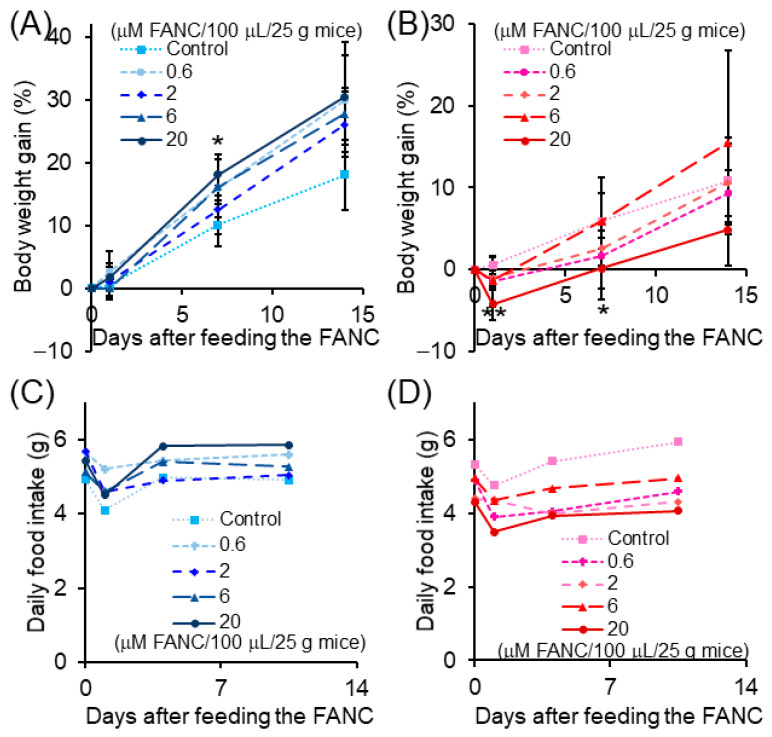
Body weight gain and daily food intake of ICR mice after feeding a single dose of FANC. Body weight gain of (**A**) male and (**B**) female mice. (**C**) Male and (**D**) female mice’s daily food intake was calculated from day 0–1, day 1–7, and day 7–14 using one mouse. Data are expressed as the mean ± SD (*n* = 5). * *p* < 0.01, ** *p* < 0.001, significantly different to control.

At the high dosage of FANC (20 μM/100 μL/25 g mice) treatment, body weight gain was increased in male mice on day 7 but decreased in female mice on days 1 and 7 compared to the control group (Figure 1A,B). However, the body weight gain all returned to normal on day 14. The daily food consumption of the high dosage FANC (20 μM/100 μL/25 g mice) treated groups was increased in male mice but decreased in females (Figure 1C,D).

**Figure 2:**
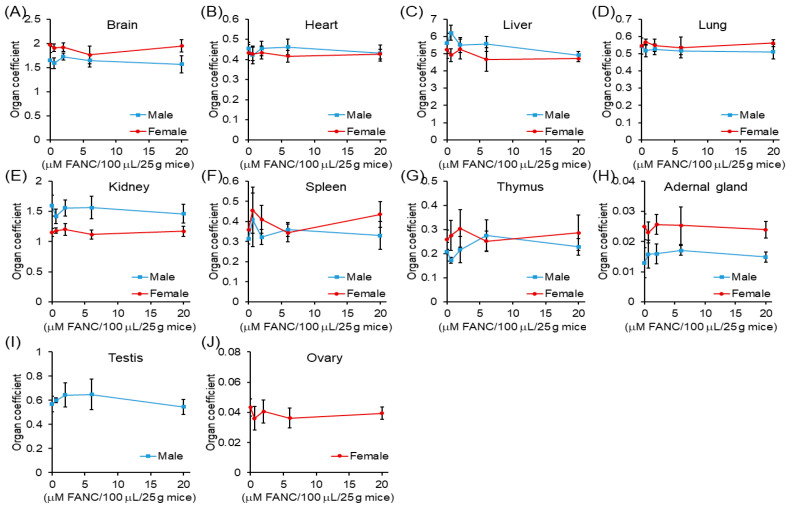
Organ coefficient changes of ICR mice after feeding a single dose of FANC for 14 days Organ coefficient = (organ weight/body weight) × 100. Data are expressed as the mean ± SD (*n* = 5).

**Figure 3:**
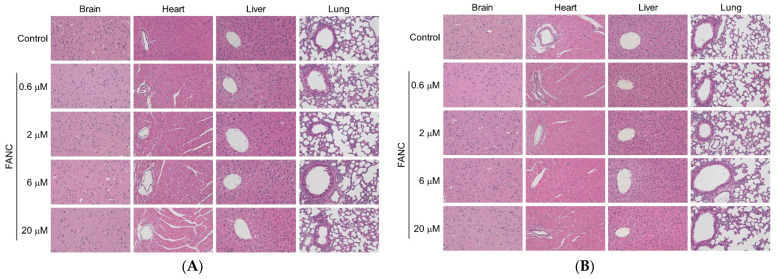

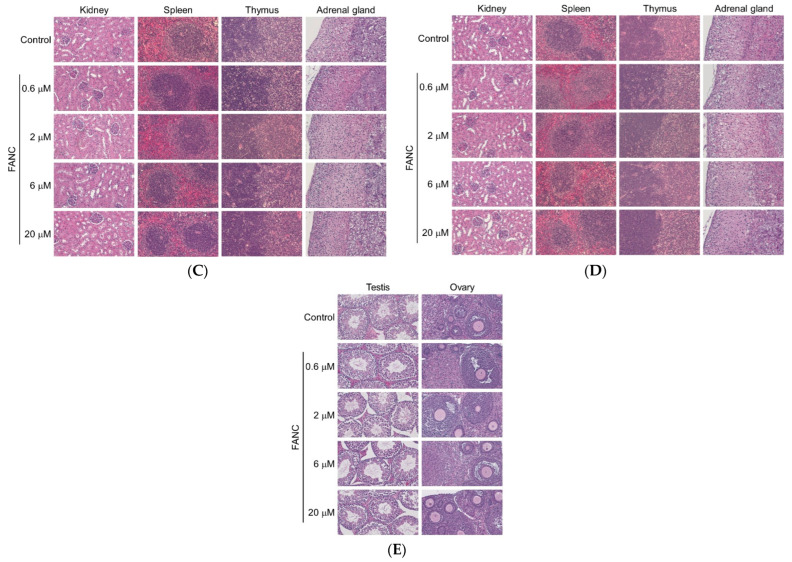
Representative H&E stained histology of tissue from ICR mice after feeding a single dose of FANC. Section of brain, heart, liver, and lung from (**A**) male and (**B**) female mice. Section of kidney, spleen, thymus, and adrenal gland from (**C**) male and (**D**) female mice. (**E**) Section of testis and ovary from mice.

At the end of the test, the anatomy revealed that the organ coefficients of the brain, heart, liver, lung, kidney, spleen, thymus, adrenal gland, testis, and ovary were not significantly different compared to that of the control groups (Figure 2). The histopathological assay serves as accessorial proof for hematological and biochemical analysis [26]. The histological structures of the brain, heart, liver, lung, kidney, spleen, thymus, and adrenal gland of ICR mice after 14 days of single-dose FANC treatment were compared with those of the controls (Figure 3A–D). No significant difference was noted in the structures of these organs between male and female mice. Single-dose FANC treatment did not induce fibrosis or inflammation in the brain, heart, liver, and lung. Single-dose FANC treatment did not induce glomeruli atrophy. The spleen displayed normal red and white (deeper purple area) pulp areas. The thymus displayed a normal cortex (deeper color area) and medulla. The histological structures of the testis and ovary were also comparable with those of controls (Figure 3E). Germ cells and seminiferous tubules in the testis and follicles in the ovary both appeared normal. No histopathological changes were observed in those organs of the control and FANC-treated groups (Figure 3).

Administration of FANC did not exert adverse effects on the hematological parameters analysis of ICR mice on days 1, 7, and 14 (Supplementary Tables S1 and S2, and Table 1). Furthermore, serum GOT, GPT, LDH, BUN, and CRE assays on the 14th day after feeding a single dose of FANC have no significant malignant changes (Table 2).

### 3.2. Subacute Toxicity Test

**Figure 4:**
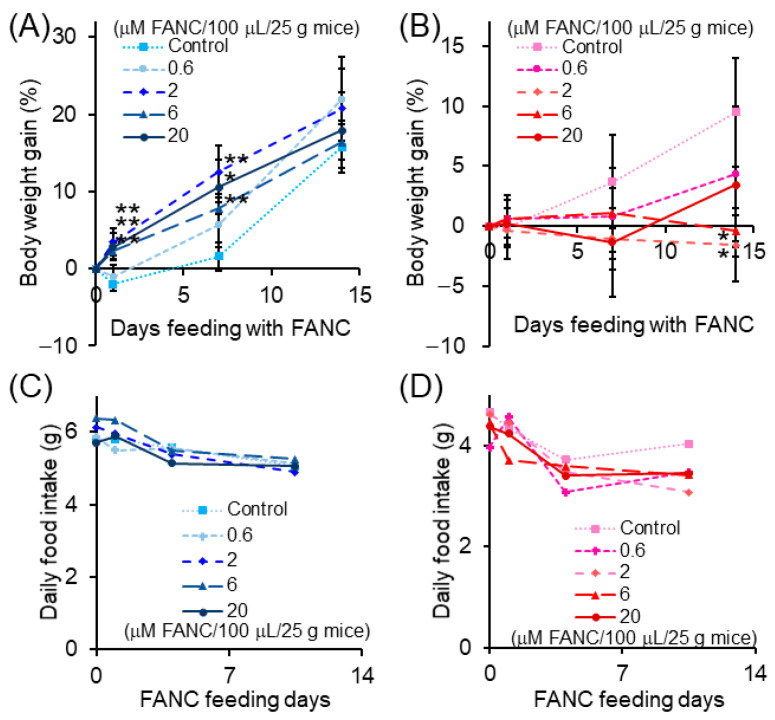
Body weight gain and daily food intake of ICR mice with daily feeding of FANC. Body weight gain of (**A**) male and (**B**) female mice. (**C**) Male and (**D**) female mice’s daily food intake was calculated from day 0–1, day 1–7, and day 7–14 using one mouse. Data are expressed as the mean ± SD (*n* = 5). * *p* < 0.01, ** *p* < 0.001, significantly different to control.

No treatment-related mortality and behavioral changes were observed in the FANC-treated as well as control animals throughout the experiment. As shown in Figure 4A, the body weight gain of male mice increased in the first weeks of the experiment compared with the control group (*p* < 0.01), but after two weeks, the body weight gain returned to normal for animals treated with 2–20 μM /100 μL/25 g mice. On day 14 of the experiment, there were significant decreases (*p* < 0.01) in body weight gain of female mice between the control and the groups that received 2 and 6 μM /100 μL/25 g mice of FANC (Figure 4B). Daily food consumption of ICR mice with daily feeding of FANC was not significantly changed in male mice but slightly decreased in females (Figure 4C,D).

**Figure 5:**
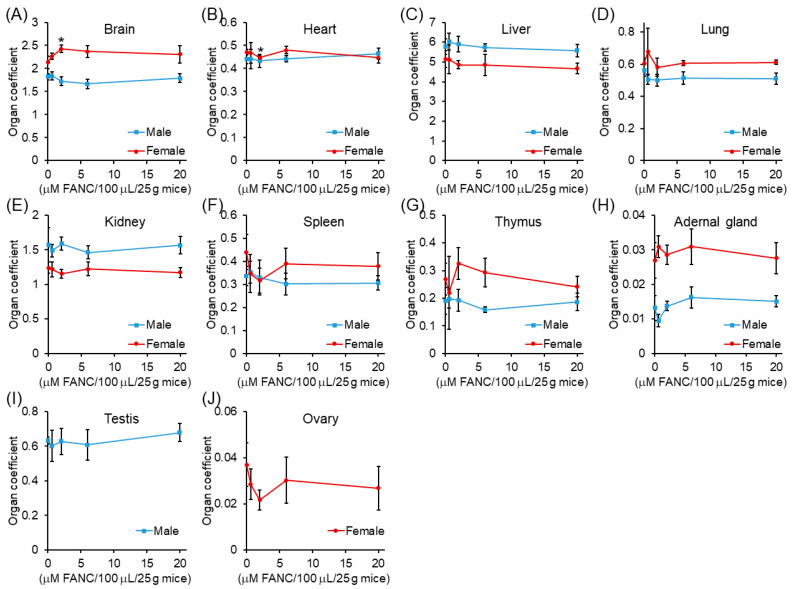
Organ coefficient changes of ICR mice with daily feeding of FANC for 14 days. Organ coefficient = (organ weight/body weight) × 100. Data are expressed as the mean ± SD (*n* = 5).

**Figure 6:**
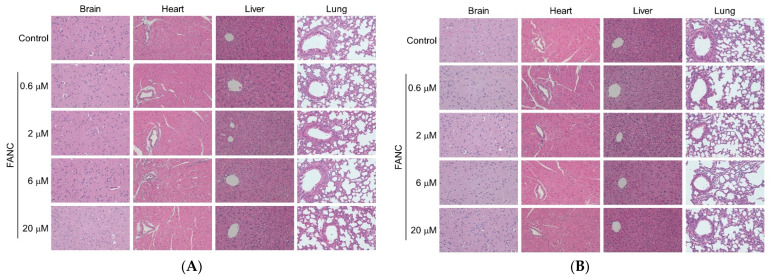

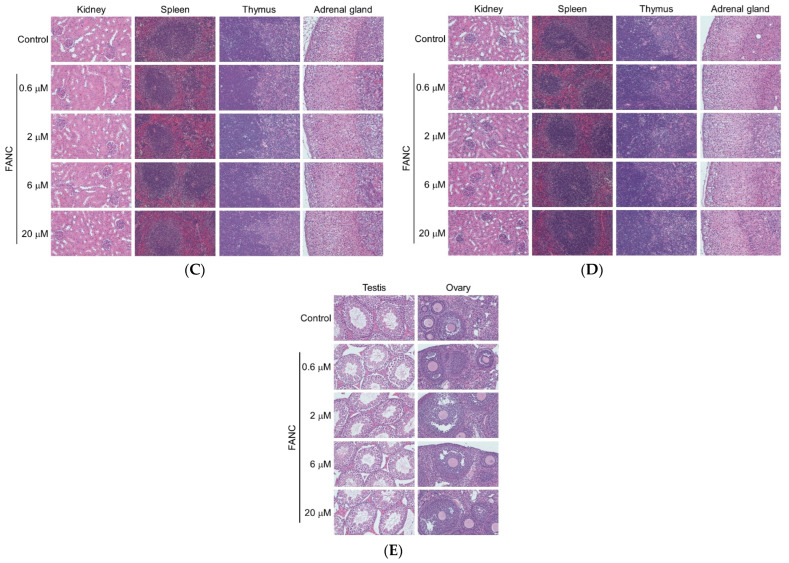
Representative H&E stained histology of tissue from ICR mice after feeding with FANC for 14 days. Section of brain, heart, liver, and lung from (**A**) male and (**B**) female mice. Section of kidney, spleen, thymus, and adrenal gland from (**C**) male and (**D**) female mice. (**E**) Section of testis and ovary from mice.

The results of the organ coefficient are presented in Figure 5. The organ coefficient of the heart, liver, kidney, testis, and ovary between males and females in experimental groups ranging from 0.6 to 20 μM /100 μL/25 g mice showed no significant difference compared with their control groups. The histological structures of the brain, heart, liver, lung, kidney, spleen, thymus, and adrenal gland of ICR mice after FANC feeding for 14 days were comparable with those of the controls (Figure 6A–D). No significant difference was found in the structures between male and female mice. Daily FANC treatment did not induce fibrosis or inflammation in the brain, heart, liver, or lung. Daily FANC treatment did not induce glomeruli atrophy. The spleen displayed normal red and white (deeper purple area) pulp areas. The thymus displayed a normal cortex (deeper color area) and medulla. The histological structures of the testis and ovary of ICR mice after FANC feeding for 14 days were comparable with those of controls (Figure 6E). Germ cells and seminiferous tubules in the testis and follicles in the ovary both appeared normal. No histopathological changes were observed in those organs of the control and FANC-treated groups (Figure 6).

Hematological parameters analysis of ICR mice after daily FANC feeding and on days 1, 7, and 14 showed no significant changes compared with the control groups (Supplementary Tables S3 and S4, and Table 3). Furthermore, serum GOT, GPT, LDH, BUN, and CRE assays after 14 days of FANC feeding showed no significant malignant changes (Table 4).

## 4. Discussion

Gold nanoclusters (Au NCs) are well-established in medicine due to their excellent biological properties. By stabilizing them with dihydrolipoic acid (DHLA), the DHLA-capped Au NCs (FANC) were synthesized [5,15]. However, there is still no report on the toxicity evaluation of FANC. The toxicology profile is required for selecting a safe dose *in vivo*. In the present study, we firstly investigated the oral acute and subacute toxicities of FANC. The mouse is one of the main mammalian species used in preclinical studies both in pharmacology and toxicology due to its small size, low life span, easy availability, and low cost.

Acute toxicity testing requires test materials to be administered to animals for a finite but short period, usually as a single exposure. The acute toxicity tests showed that, at the tested doses, no behavioral changes, toxic symptoms, or death were observed in all mice. Therefore, it is assumed that the LD50 of FANC was above 20 μM/100 μL/25 g mice. The dose–response evidence of possible health risks was provided after repeated administrations for 14 days during the subacute toxicity test. Thus, four different FANC doses (0.6, 2, 6, or 20 μM/100 μL/25 g mice) were administered to two sexes of animals in the present study for toxicological evaluations. During the experiment period, the weight gain of high-dose treatment (6 and 20 μM/100 μL/25 g mice) groups was significantly increased in males in the first week but returned to normal after two weeks. However, the weight gain of intermediary dose treatment (2 and 6 μM/100 μL/25 g mice) groups decreased significantly in the female mice after two weeks. The organs were removed and weighed at the end of the test periods, and no significant difference was observed in all organ weights compared to the control groups. Though it is not dosage-dependent in the present study, the changes in body weight may be due to either food consumption or organ injuries caused by the test substance, indicating that FANC needs to be further evaluated for its possible side effects. Therefore, hematological, serum biochemical, and histopathological parameters are necessary to assess the toxicity of FANC and determine its toxic effects on organs.

The hematopoietic parameters are the most sensitive to the toxic effects of the substances and are used for assessing physiological and pathological status in humans and animals [27]. The RBC and HCT values were decreased significantly in (0.6 μM/100 μL/25 g mice) the treated female mice in the acute test. However, the RBC and HCT values were increased significantly in (2 μM/100 μL/25 g mice) the treated male mice in the subacute test. The RDW value was also significantly decreased in the male mice of (2 and 20 μM/100 μL/25 g mice) the treated group in the subacute test. These changes suggested that the FANC might affect the erythropoiesis, morphology, and osmotic fragility of RBC, indicating that the liver and kidney tissues are damaged [28].

However, there is no dose–response evidence, and the changes should be further evaluated by biochemical analysis and histopathological examination results. The changes in the WBC, LYM%, LYM#, and coefficient of the spleen were due to influences on immune function. PLT number changes can roughly evaluate the effect of treatment on hemostasis. In the present study, it was shown that all tested dosages of FANC treatment had no toxicity on the immune system and platelet number in both sexes of mice. Though some hematological parameters of the FANC treatment groups were significantly different from the control, they were not dose-dependent and still in the normal range [29]. There was also no alteration in the general function of the test organism. We consider that the differences in values are not related to treatment, but were caused by chance deviations.

Evaluating glucose metabolism and protein synthesis can assess liver function, and GOT, GPT, LDH, and gamma-glutamyltransferase are known as sensitive biomarkers of hepatocellular function [30]. Rising GPT and GOT levels are an index for the diagnosis of liver damage with hepatitis or hepatic toxicity [31]. Though the GPT of the (2 μM/100 μL/25 g mice) male group decreased remarkably in the subacute test, it is suggested that FANC should not possess toxicity to the liver tissue and result in liver injuries. The increase in CRE and BUN indicated renal function injury [32]. The BUN and CRE contents in the tests presented no difference in comparison to the control group, showing that FANC is not toxic to the kidney.

Oral administration of FANC may cause less body weight gain in female ICR mice than in the controls. However, there is no obvious dose–response effect in body weight decreases, and the decreasing level was below the threshold of concern (10%). As shown in the results, the microscopic observation revealed that no pathological changes in the brain, heart, liver, lung, kidney, spleen, thymus, adrenal gland, testis, and ovary were observed in the experimental animals after administration of all doses of FANC in both acute and subacute toxicity tests. Animal behavior, body weight, organ coefficient, daily food consumption, hematological profile, serum biochemistry, and histopathological analysis showed no malignant changes at single and repeated doses. The no observed adverse effect level (NOAEL) is considered 20 μM/100 μL/25 g mice in male and female ICR mice.

Compared with traditional nanoparticles (NPs), the size of gold nanoclusters (NCs) is smaller. Unlike large-size gold nanoparticles (5–200 nm), sizes below 1.4–4 nm gastrointestinal uptake were more readily available [3,33]. Typical thiol-protected Au NCs with the size of 1–2 nm have a stronger enhanced permeability and retention effect (EPR) than larger-size particles [7]. The FANC we used in this study was 1.56 ± 0.3 nm in diameter [15], which may have had a better gastrointestinal uptake rate.

The toxicity of gold nanoparticles was dependent on size, and the particle size of 8–37 nm was lethal for mice at 8 mg/kg/week [29]. However, a size below 5 nm did not show any harmful effects at the same concentration [29]. The gold nanocluster smaller than 2 nm was easier to clear renally and avoided high-level accumulation and toxicity [34,35]. The previous study also displayed the low toxicity of gold nanoclusters at small sizes [7,36]. The FANC we used in this study was 1.56 ± 0.3 nm in diameter per gold nanocluster [15], and we did not observe morbidity. However, chronic toxicity and organ accumulation still need further research to discuss.

For the toxicity test, the highest dose for FANC was chosen to be 20 μM/100 μL/25 g mice because the synthesized FANC’s maximum concentration is 20 μM. In addition, this concentration was more than 2.72 times higher than the effective dose in the previous study [25]. The FANC administration route used in this study was orally by gavage, and oral administration was usually widely accepted by users.

## Figures and Tables

**Table 1:** Changes of hematological parameters of ICR mice after feeding the FANC for 14 days.

**Male**	**0**	**0.6**	**2**	**6**	**20**
RBC	6.3 ± 0.3	6.2 ± 0.2	6.2 ± 0.4	6.1 ± 0.1	6.2 ± 0.5
HCT	34.5 ± 1.3	34.7 ± 1.2	33.9 ± 1.9	33.3 ± 1.2	34.0 ± 2.8
RDW	2.6 ± 12.2	12.1 ± 1.1	7.3 ± 10.2	7.2 ± 10.1	11.5 ± 0.4
WBC	4.0 ± 1.2	5.4 ± 2.3	3.7 ± 0.5	3.5 ± 1.3	3.0 ± 0.3
LYM%	78.2 ± 2.7	75.6 ± 7.9	81.4 ± 9.2	73.6 ± 2.9	73.0 ± 10.6
LYM#	3.2 ± 0.9	4.2 ± 2.2	3.1 ± 0.7	2.6 ± 1.0	2.2 ± 0.2
PLT	910.4 ± 87.3	896.8 ± 54.3	830.8 ± 69.7	870.6 ± 119.9	949.2 ± 232.3
PDW	5.8 ± 0.4	5.9 ± 0.3	5.8 ± 0.2	5.8 ± 0.2	5.7 ± 0.2
MPV	5.2 ± 0.2	5.4 ± 0.3	5.2 ± 0.1	5.3 ± 0.2	5.2 ± 0.1
P-LCR	2.1 ± 0.2	2.6 ± 1.4	2.1 ± 0.7	2.0 ± 0.6	2.1 ± 0.5
**Female**	**0**	**0.6**	**2**	**6**	**20**
RBC	6.7 ± 0.3	5.9 ± 0.1 **	6.4 ± 0.3	6.6 ± 0.3	6.6 ± 0.2
HCT	35.9 ± 1.9	31.4 ± 1.2 *	34.2 ± 2.2	35.3 ± 1.4	34.7 ± 1.5
RDW	7.4 ± 10.2	12.3 ± 0.9	7.9 ± 10.6	12.2 ± 1.0	12.5 ± 0.6
WBC	3.9 ± 0.7	3.4 ± 0.6	4.5 ± 1.0	4.5 ± 2.2	4.2 ± 1.2
LYM%	80.4 ± 3.8	82.5 ± 9.3	83.7 ± 6.8	81.8 ± 4.8	78.6 ± 10.3
LYM#	3.3 ± 0.5	2.8 ± 0.8	3.8 ± 0.9	3.7 ± 1.9	3.3 ± 1.4
PLT	801.2 ±192.0	705.4 ± 225.7	747.8 ± 153.7	906.0 ± 131.8	768.8 ± 181.2
PDW	5.8 ± 0.4	5.8 ± 0.2	5.6 ± 0.1	5.8 ± 0.4	5.8 ± 0.1
MPV	5.2 ± 0.2	5.3 ± 0.1	5.1 ± 0.0	5.3 ± 0.4	5.2 ± 0.1
P-LCR	2.0 ± 0.8	2.4 ± 0.6	1.8 ± 0.4	3.0 ± 2.1	1.9 ± 0.2

RBC: red blood cell (10^6^/μL); HCT: hematocrit (%); RDW: red blood cell distribution width (%); WBC: white blood cell (10^3^/μL); LYM%: lymphocyte percentage (%); LYM#: lymphocyte count (10^3^/μL); PLT: platelets (10^3^/μL); PDW: platelet distribution width (fL); MPV: mean platelet volume (fL); P-LCR: platelet large cell ratio (%). Data are expressed as the mean ± SD (*n* = 5). * *p* < 0.01, ** *p* < 0.001, significantly different to control.

**Table 2:** Serum biochemistry changes in liver and renal function of ICR mice after feeding a single dose of FANC for 14 days.

**Male**	**GOT**	**GPT**	**LDH**	**BUN**	**CRE**
**(μM)**	**(U/L)**	**(U/L)**	**(U/L)**	**(mg/dL)**	**(mg/dL)**
0	36.2 ± 3.9	33.4 ± 10.7	479.0 ± 210.7	19.6 ± 3.4	0.2 ± 0.1
0.6	34.3 ± 5.1	32.3 ± 8.8	275.5 ± 115.7	23.2 ± 1.3	0.3 ± 0.1
2	32.6 ± 4.7	28.2 ± 10.5	310.0 ± 152.1	22.7 ± 5.1	0.2 ± 0.1
6	38.4 ± 6.1	29.4 ± 3.0	373.2 ± 283.7	25.7 ± 2.9	0.3 ± 0.1
20	48.6 ± 9.8	34.8 ± 7.4	515.8 ± 238.1	20.5 ± 2.3	0.2 ± 0.0
**Female**	**GOT**	**GPT**	**LDH**	**BUN**	**CRE**
**(μM)**	**(U/L)**	**(U/L)**	**(U/L)**	**(mg/dL)**	**(mg/dL)**
0	50.6 ± 8.6	32.4 ± 6.5	363.4 ± 93.6	23.7 ± 2.2	0.1 ± 0.0
0.6	46.4 ± 8.1	30.4 ± 4.9	369.8 ± 155.3	21.8 ± 1.9	0.1 ± 0.0
2	40.8 ± 8.5	32.8 ± 8.4	260.4 ± 97.0	21.4 ± 2.6	0.1 ± 0.0
6	41.4 ± 9.0	32.8 ± 10.0	257.4 ± 116.1	23.4 ± 4.4	0.1 ± 0.1
20	46.0 ± 10.4	31.4 ± 9.3	302.2 ± 77.9	23.0 ± 3.5	0.1 ± 0.0

GOT: glutamic-oxaloacetic transaminase; GPT: glutamic-pyruvic transaminase; LDH: lactate dehydrogenase; BUN: blood urea nitrogen; CRE: creatinine. Data are expressed as the mean ± SD (*n* = 5).

**Table 3:** Changes of hematological parameters of ICR mice feeding with FANC for 14 days.

**Male**	**0**	**0.6**	**2**	**6**	**20**
RBC	5.9 ± 0.3	5.8 ± 0.3	6.7 ± 0.3	5.9 ± 0.5	6.7 ± 0.6
HGB	11.0 ± 0.5	11.2 ± 0.4	11.9 ± 0.9	10.9 ± 0.5	12.2 ± 0.8
HCT	31.0 ± 1.6	31.0 ± 2.2	35.3 ± 0.6 *	31.2 ± 3.2	35.2 ± 3.0
MCV	52.7 ± 0.8	53.4 ± 1.2	53.9 ± 0.5	53.4 ± 1.0	52.6 ± 2.1
MCH	18.7 ± 1.3	19.4 ± 1.2	17.9 ± 0.9	18.7 ± 1.8	18.3 ± 1.3
MCHC	35.5 ± 2.5	36.4 ± 3.0	33.2 ± 1.6	35.1 ± 3.9	34.7 ± 1.3
RDW	15.5 ± 0.8	15.2 ± 2.2	13.1 ± 0.8 *	14.4 ± 2.0	13.7 ± 0.9
WBC	4.2 ± 0.9	4.1 ± 0.6	3.4 ± 1.1	3.7 ± 1.1	4.5 ± 0.5
LYM%	78.1 ± 5.3	86.5 ± 6.9	79.0 ± 6.4	81.8 ± 3.2	80.9 ± 1.4
LYM#	3.3 ± 0.9	3.6 ± 0.5	2.7 ± 0.9	3.1 ± 1.0	3.6 ± 0.4
PLT	582.4 ± 110.3	721.4 ± 168.6	739.0 ± 82.9	653.0 ± 242.8	699.4 ± 131.9
**Female**	**0**	**0.6**	**2**	**6**	**20**
RBC	6.8 ± 0.3	7.0 ± 0.4	6.4 ± 0.5	6.5 ± 0.3	6.6 ± 0.2
HGB	11.3 ± 1.1	12.2 ± 0.7	10.4 ± 0.9	11.0 ± 0.6	10.5 ± 0.5
HCT	37.5 ± 2.9	38.5 ± 2.3	34.1 ± 2.9	35.2 ± 1.5	35.2 ± 1.5
MCV	54.8 ± 2.6	54.8 ± 1.9	53.3 ± 2.0	54.2 ± 1.5	53.3 ± 1.0
MCH	16.4 ± 1.1	17.4 ± 0.9	16.3 ± 0.8	16.9 ± 0.9	15.9 ± 0.4
MCHC	30.0 ± 1.0	31.7 ± 0.7	30.6 ± 0.5	31.2 ± 1.1	29.8 ± 0.5
RDW	11.9 ± 0.5	12.0 ± 0.5	11.7 ± 0.5	11.1 ± 0.5	11.7 ± 0.5
WBC	4.2 ± 1.4	3.9 ± 0.9	3.7 ± 1.5	3.0 ± 1.0	3.8 ± 0.9
LYM%	84.1 ± 5.6	81.5 ± 1.4	81.4 ± 4.9	79.3 ± 7.1	83.2 ± 3.3
LYM#	3.6 ± 1.3	3.2 ± 0.7	3.0 ± 1.3	2.4 ± 0.9	3.1 ± 0.7
PLT	733.0 ± 171.2	651.8 ± 77.4	736.4 ± 168.9	797.0 ± 63.8	783.6 ± 160.2

RBC: red blood cell (10^6^/μL); HGB: hemoglobin (g/dL); HCT: hematocrit (%); MCV: mean corpuscular volume (fL); MCH: mean corpuscular hemoglobin (pg); MCHC: mean corpuscular hemoglobin concentration (g/dL); RDW: red blood cell distribution width (%); WBC: white blood cell (10^3^/μL); LYM%: lymphocyte percentage (%); LYM#: lymphocyte count (10^3^/μL); PLT: platelets (10^3^/μL). Data are expressed as the mean ± SD (*n* = 5). * *p* < 0.01, significantly different to control.

**Table 4:** Serum biochemistry changes in liver and renal function of ICR mice feeding with FANC for 14 days.

**Male**	**GOT**	**GPT**	**LDH**	**BUN**	**CRE**
**(μM)**	**(U/L)**	**(U/L)**	**(U/L)**	**(mg/dL)**	**(mg/dL)**
0	35.8 ± 6.8	19.0 ± 3.0	295.0 ± 167.7	19.1 ± 4.3	0.2 ± 0.1
0.6	41.0 ± 8.2	24.2 ± 10.1	404.5 ± 94.1	23.2 ± 0.9	0.2 ± 0.1
2	33.8 ± 5.4	14.0 ± 2.3	295.2 ± 120.1	21.4 ± 1.8	0.1 ± 0.0
6	37.4 ± 2.5	15.4 ± 3.4	216.8 ± 47.9	22.7 ± 6.1	0.2 ± 0.1
20	36.4 ± 4.7	16.4 ± 2.9	239.3 ± 65.4	19.9 ± 3.7	0.2 ± 0.1
**Female**	**GOT**	**GPT**	**LDH**	**BUN**	**CRE**
**(μM)**	**(U/L)**	**(U/L)**	**(U/L)**	**(mg/dL)**	**(mg/dL)**
0	44.0 ± 5.6	21.0 ± 3.1	226.2 ± 47.4	20.3 ± 4.0	0.1 ± 0.0
0.6	47.3 ± 9.1	23.5 ± 14.7	280.8 ± 176.1	24.0 ± 5.8	0.1 ± 0.0
2	47.8 ± 6.2	14.8 ± 1.6 *	335.8 ± 100.0	19.7 ± 1.4	0.1 ± 0.0
6	54.0 ± 12.7	17.0 ± 6.7	215.0 ± 94.6	20.8 ± 1.6	0.1 ± 0.0
20	46.8 ± 4.7	20.6 ± 5.8	197.8 ± 72.7	23.0 ± 2.3	0.1 ± 0.0

GOT: glutamic-oxaloacetic transaminase; GPT: alanine aminotransferase; LDH: lactate dehydrogenase; BUN: blood urea nitrogen; CRE: creatinine. Data are expressed as the mean ± SD (*n* = 5). * *p* < 0.01, significantly different to control.

## Data Availability

Not applicable.

## Supplementary Materials

The following supporting information can be downloaded at: https://www.mdpi.com/article/10.3390/nano12213868/s1, Table S1: Changes of hematological parameters of ICR mice after feeding the FANC for 1 day. Table S2: Changes of hematological parameters of ICR mice after feeding the FANC for 7 day. Table S3: Changes of hematological parameters of ICR mice feeding with FANC for 1 day. Table S4: Changes of hematological parameters of ICR mice feeding with FANC for 7 day.
