# Supplementary material for: Acute and Subacute Toxicity of Fluorescent Gold Nanoclusters Conjugated with α-Lipoic Acid
Source: Nanomaterials (Basel). 2022 Nov 2;12(21):3868. doi: 10.3390/nano12213868 (PMC9654421; doi:10.3390/nano12213868)
Supplement: Supplementary file 1 [file nanomaterials-12-03868-s001.zip › nanomaterials-1948182-supplementary.pdf]

Supplementary Table S1. Changes of hematological parameters of ICR mice after feeding the FANC for 1 day

Male	0	0.6	2	6	20
RBC	6.7 ± 0.4	6.3 ± 0.5	6.7 ± 0.6	6.5 ± 0.4	6.7 ± 0.3
HCT	38.1 ± 3.2	36.2 ± 3.6	37.6 ± 1.8	36.5 ± 2.1	38.1 ± 1.6
RDW	12.5 ± 1.0	13.2 ± 0.7	13.7 ± 0.6	13.5 ± 0.4	13.2 ± 1.3
WBC	7.9 ± 5.0	7.8 ± 5.0	7.3 ± 4.4	6.4 ± 2.2	7.5 ± 4.7
LYM%	67.5 ± 8.5	71.1 ± 8.0	79.4 ± 10.9	69.5 ± 8.9	76.4 ± 16.6
LYM#	5.0 ± 4.3	5.6 ± 3.9	5.8 ± 3.3	4.5 ± 2.0	5.7 ± 3.5
PLT	589.0 ± 387.9	442.8 ± 301.1	898.8 ± 41.6	656.8 ± 253.3	685.8 ± 373.9
PDW	6.7 ± 1.1	7.5 ± 1.5	6.6 ± 0.6	6.1 ± 0.4	6.4 ± 0.7
MPV	5.7 ± 0.5	6.1 ± 0.5	5.7 ± 0.3	5.5 ± 0.3	5.6 ± 0.5
P-LCR	4.1 ± 3.3	6.2 ± 3.2	3.4 ± 1.6	3.0 ± 1.3	3.4 ± 1.9

Female	0	0.6	2	6	20
RBC	6.8 ± 0.6	6.9 ± 0.3	7.3 ± 0.7	7.3 ± 0.6	6.2 ± 3.7
HCT	37.0 ± 2.6	38.3 ± 2.1	40.4 ± 4.7	40.2 ± 3.2	35.1 ± 18.3
RDW	13.2 ± 0.8	12.9 ± 0.8	12.6 ± 0.5	12.6 ± 0.8	12.6 ± 1.0
WBC	9.0 ± 2.0	8.5 ± 2.8	9.2 ± 1.7	8.7 ± 1.5	8.5 ± 4.7
LYM%	75.0 ± 11.4	77.8 ± 12.2	69.4 ± 13.3	71.7 ± 10.4	64.0 ± 16.7
LYM#	6.9 ± 2.0	6.9 ± 3.0	6.5 ± 2.4	6.2 ± 1.3	6.5 ± 0.7
PLT	457.2 ± 229.0	388.0 ± 238.8	471.2 ± 284.9	428.6 ± 357.9	297.8 ± 209.3
PDW	6.6 ± 0.5	6.3 ± 0.2	5.9 ± 0.2	6.3 ± 0.4	7.6 ± 1.7
MPV	5.7 ± 0.2	5.5 ± 0.2	5.4 ± 0.1	5.6 ± 0.1	5.8 ± 0.4
P-LCR	3.6 ± 1.0	2.3 ± 0.7	2.4 ± 0.7	3.3 ± 0.9	3.4 ± 3.4

RBC: red blood cell (10^6/μL); HCT: hematocrit (%); RDW: red blood cell distribution width (%); WBC: white blood cell (10^3/μL); LYM%: lymphocyte percentage (%); LYM#: lymphocyte count (10^3/μL); PLT: platelets (10^3/μL); PDW: platelet distribution width (fL); MPV: mean platelet volume (fL); P-LCR: platelet large cell ratio (%). Data are expressed as the mean ± SD (n=5).

Supplementary Table S2. Changes of hematological parameters of ICR mice after feeding the FANC for 7 day

Male	0	0.6	2	6	20
RBC	6.9 ± 0.3	6.7 ± 0.6	6.7 ± 0.4	6.4 ± 0.4	6.6 ± 0.4
HCT	38.1 ± 1.1	37.6 ± 2.6	36.7 ± 1.5	35.2 ± 1.7	36.8 ± 2.6
RDW	12.1 ± 0.6	12.5 ± 0.8	12.3 ± 0.6	12.5 ± 0.4	12.6 ± 0.7
WBC	5.7 ± 3.0	11.1 ± 2.5	9.5 ± 3.7	7.1 ± 1.8	10.5 ± 2.8
LYM%	65.5 ± 18.0	65.8 ± 6.5	75.4 ± 16.3	74.3 ± 7.8	68.1 ± 8.6

LYM#	3.9 ± 0.5	6.4 ± 1.3	5.2 ± 1.0	6.8 ± 1.3	7.1 ± 1.5
PLT	374.4 ± 416.8	409.6 ± 136.8	808.0 ± 324.7	442.2 ± 363.8	448.2 ± 229.5
PDW	5.9 ± 0.1	8.7 ± 2.0	6.9 ± 1.6	5.9 ± 0.0	7.0 ± 1.3
MPV	5.3 ± 0.1	6.3 ± 0.4	5.8 ± 0.6	5.5 ± 0.2	5.8 ± 0.3
P-LCR	2.2 ± 0.1	7.6 ± 2.6	4.7 ± 3.7	3.1 ± 1.6	4.8 ± 1.6

Female	0	0.6	2	6	20
RBC	7.4 ± 0.6	8.1 ± 0.4	7.7 ± 0.2	8.0 ± 0.1	7.3 ± 0.3
HCT	40.0 ± 2.0	42.8 ± 2.3	32.7 ± 19.4	43.1 ± 0.7	39.9 ± 1.8
RDW	13.1 ± 0.9	12.6 ± 0.5	12.4 ± 0.8	12.2 ± 0.3	12.7 ± 0.5
WBC	9.8 ± 1.4	9.0 ± 2.7	6.3 ± 1.7	9.7 ± 1.8	10.3 ± 1.5
LYM%	66.3 ± 6.3	69.8 ± 6.2	69.8 ± 17.9	59.4 ± 15.7	77.0 ± 4.8
LYM#	6.4 ± 0.3	5.6 ± 1.2	4.3 ± 1.1	5.4 ± 1.5	7.6 ± 1.6
PLT	221.2 ±151.8	399.8 ± 314.5	434.3 ± 437.9	331.0 ± 282.3	350.0 ± 233.9
PDW	8.6 ± 0.8	6.6 ± 1.0	6.0 ± 0.0	6.6 ± 0.8	8.1 ± 2.2
MPV	6.4 ± 0.4	5.5 ± 0.4	5.4 ± 0.1	5.7 ± 0.5	6.3 ± 0.8
P-LCR	6.8 ± 4.8	2.8 ± 1.7	1.9 ± 1.6	3.9 ± 2.7	7.0 ± 4.8

RBC: red blood cell (10^6/μL); HCT: hematocrit (%); RDW: red blood cell distribution width (%); WBC: white blood cell (10^3/μL); LYM%: lymphocyte percentage (%); LYM#: lymphocyte count (10^3/μL); PLT: platelets (10^3/μL); PDW: platelet distribution width (fL); MPV: mean platelet volume (fL); P-LCR: platelet large cell ratio (%). Data are expressed as the mean ± SD (n=5).

Supplementary Table S3. Changes of hematological parameters of ICR mice feeding with FANC for 1 day

Male	0	0.6	2	6	20
RBC	6.8 ± 0.2	6.7 ± 0.3	6.6 ± 0.3	6.4 ± 0.3	6.8 ± 0.3
HGB	11.9 ± 0.7	11.6 ± 0.7	11.7 ± 0.4	11.4 ± 0.1	11.7 ± 0.1
HCT	39.6 ± 1.7	37.8 ± 2.3	37.4 ± 1.5	36.1 ± 1.1	37.4 ± 1.7
MCV	58.0 ± 1.5	56.2 ± 1.0	57.1 ± 1.1	56.4 ± 1.5	55.2 ± 2.0
MCH	17.5 ± 0.7	17.3 ± 0.5	17.9 ± 0.3	17.8 ± 0.8	17.2 ± 0.7
MCHC	30.2 ± 0.9	30.8 ± 0.6	31.4 ± 0.6	31.5 ± 0.9	31.2 ± 1.3
RDW	14.3 ± 1.1	13.9 ± 0.3	12.7 ± 0.7	13.2 ± 0.6	13.4 ± 0.8
WBC	7.0 ± 2.2	4.6 ± 1.2	5.6 ± 2.9	4.7 ± 0.9	7.1 ± 1.4
LYM%	70.1 ± 8.3	74.8 ± 9.5	74.7 ± 12.6	76.2 ± 4.7	77.5 ± 10.4
LYM#	5.0 ± 2.1	3.4 ± 1.2	4.0 ± 1.8	3.6 ± 0.8	5.4 ± 0.6
PLT	711.2 ± 117.8	812.2 ± 112.2	553.6 ± 321.7	834.6 ± 129.6	788.0 ± 147.8

Female	0	0.6	2	6	20

RBC	6.7 ± 0.5	7.0 ± 0.5	7.2 ± 0.3	5.0 ± 0.3	5.3 ± 0.2
HGB	11.9 ± 1.2	12.1 ± 0.6	12.3 ± 0.8	13.2 ± 0.9	12.7 ± 0.4
HCT	37.2 ± 2.2	38.4 ± 2.2	38.6 ± 1.6	28.0 ± 1.3	29.2 ± 0.8
MCV	55.2 ± 2.5	54.8 ± 1.8	53.8 ± 1.7	55.8 ± 0.9	32.4 ± 49.7
MCH	17.7 ± 1.2	17.4 ± 1.0	17.0 ± 0.6	26.3 ± 1.2	23.9 ± 1.3
MCHC	32.0 ± 1.4	31.5 ± 0.9	32.0 ± 0.7	47.1 ± 1.7	43.5 ± 1.9
RDW	13.3 ± 0.4	15.3 ± 1.1	14.7 ± 1.1	22.7 ± 2.3	19.8 ± 1.4
WBC	8.4 ± 3.4	8.6 ± 3.2	7.7 ± 3.1	6.0 ± 2.8	8.3 ± 0.7
LYM%	73.8 ± 9.8	71.8 ± 11.6	66.0 ± 11.7	58.6 ± 7.5	71.0 ± 12.9
LYM#	6.1 ± 2.3	6.1 ± 2.3	5.0 ± 2.0	3.5 ± 1.6	5.9 ± 1.4
PLT	389.4 ± 202.1	620.8 ± 183.8	421.0 ± 281.7	636.4 ± 70.2	579.8 ± 107.7

RBC: red blood cell ($10^6/\mu L$); HGB: hemoglobin (g/dL); HCT: hematocrit (%); MCV: mean corpuscular volume (fL); MCH: mean corpuscular hemoglobin (pg); MCHC: mean corpuscular hemoglobin concentration (g/dL); RDW: red blood cell distribution width (%); WBC: white blood cell ($10^3/\mu L$); LYM%: lymphocyte percentage (%); LYM#: lymphocyte count ($10^3/\mu L$); PLT: platelets ($10^3/\mu L$). Data are expressed as the mean ± SD (n=5).

Supplementary Table S4. Changes of hematological parameters of ICR mice feeding with FANC for 7 day

Male	0	0.6	2	6	20
RBC	7.1 ± 0.2	7.2 ± 0.3	7.5 ± 0.4	7.3 ± 0.1	7.5 ± 0.1
HGB	12.2 ± 0.3	12.3 ± 0.5	13.1 ± 0.4	13.1 ± 0.4	12.8 ± 0.3
HCT	38.4 ± 0.9	39.5 ± 1.7	41.7 ± 2.2	39.9 ± 1.2	40.8 ± 1.4
MCV	54.3 ± 0.4	55.1 ± 0.9	55.2 ± 0.5	55.0 ± 1.3	53.7 ± 1.3
MCH	17.1 ± 0.4	17.1 ± 0.2	17.8 ± 0.3	18.0 ± 0.6	16.9 ± 0.4
MCHC	31.5 ± 0.6	31.0 ± 0.4	32.1 ± 0.7	32.8 ± 1.2	31.6 ± 0.6
RDW	12.5 ± 0.8	12.6 ± 0.6	12.7 ± 0.6	13.5 ± 0.6	13.5 ± 0.8
WBC	6.5 ± 2.2	7.2 ± 0.9	7.9 ± 0.4	8.5 ± 2.6	9.0 ± 1.0
LYM%	66.5 ± 6.8	72.1 ± 16.6	63.6 ± 11.8	74.1 ± 5.3	62.9 ± 11.9
LYM#	4.3 ± 1.5	5.1 ± 0.9	5.0 ± 0.9	6.3 ± 2.1	5.7 ± 1.6
PLT	614.2 ± 153.0	525.6 ± 281.1	347.0 ± 145.6	537.6 ± 176.6	400.0 ± 125.5

Female	0	0.6	2	6	20
RBC	6.9 ± 1.2	7.7 ± 0.4	7.7 ± 1.0	7.8 ± 0.6	8.1 ± 0.5
HGB	14.1 ± 1.0	13.9 ± 1.0	13.9 ± 1.2	13.8 ± 0.7	14.0 ± 0.6
HCT	40.0 ± 6.9	42.9 ± 2.9	42.5 ± 6.3	44.6 ± 2.9	46.1 ± 2.1
MCV	57.7 ± 1.6	55.7 ± 1.5	54.8 ± 1.7	57.0 ± 1.2	56.9 ± 1.4

MCH	21.4 ± 5.8	18.1 ± 0.7	18.1 ± 1.0	17.6 ± 0.7	17.3 ± 0.9
MCHC	36.4 ± 9.5	32.5 ± 0.6	33.1 ± 2.7	30.9 ± 0.8	30.5 ± 0.8
RDW	14.9 ± 5.9	13.7 ± 1.5	14.4 ± 2.2	11.3 ± 0.6	11.3 ± 0.6
WBC	5.8 ± 3.6	8.2 ± 1.6	6.5 ± 2.0	4.1 ± 1.5	5.9 ± 2.3
LYM%	76.5 ± 8.3	79.5 ± 12.9	73.5 ± 14.0	67.1 ± 5.5	67.0 ± 8.5
LYM#	4.2 ± 2.2	6.5 ± 1.8	4.7 ± 1.7	2.8 ± 1.1	3.8 ± 1.1
PLT	656.8 ± 130.1	558.8 ± 174.7	570.8 ± 132.4	711.8 ± 115.8	615.6 ± 140.8

RBC: red blood cell (10^6/μL); HGB: hemoglobin (g/dL); HCT: hematocrit (%); MCV: mean corpuscular volume (fL); MCH: mean corpuscular hemoglobin (pg); MCHC: mean corpuscular hemoglobin concentration (g/dL); RDW: red blood cell distribution width (%); WBC: white blood cell (10^3/μL); LYM%: lymphocyte percentage (%); LYM#: lymphocyte count (10^3/μL); PLT: platelets (10^3/μL). Data are expressed as the mean ± SD (n=5).
